# A Community-Based Participatory Research Program to Assess Community Lead Exposure Risk: Establishing Research Priorities

**DOI:** 10.21203/rs.3.rs-6279421/v1

**Published:** 2025-03-28

**Authors:** Jocelyn Zavala Garcia, Cículo de Salud CCATE, Nina Ali, Obed Arango, Caitlin Brady, Rosalba Esquivel-Cote, Steven Goldsmith, Holly Link, Diana Lugo, Serena Matos, Ruth McDermott-Levy, Kabindra Shakya, Daniel Jackson Smith

**Affiliations:** Villanova University; Centro de Cultura, Arte, Trabajo y Educación; University at Buffalo, State University of New York; Centro de Cultura, Arte, Trabajo y Educación; Centro de Cultura, Arte, Trabajo y Educación; Centro de Cultura, Arte, Trabajo y Educación; Villanova University; Villanova University; Centro de Cultura, Arte, Trabajo y Educación; Villanova University; Villanova University; Villanova University; University at Buffalo, State University of New York

**Keywords:** Health Promotion, Behaviors and Disease Prevention, Social Determinants of Health, Family, Lead Exposure Risk

## Abstract

**Background:**

Lead is an environmental health hazard that disproportionately impacts communities of color across the United States. Recent incidents of widespread lead exposure have been linked to aging infrastructure, historical land use, and challenges in lead remediation.

**Purpose:**

To determine community research priorities for a subsequent lead exposure assessment in a primarily Latino community.

**Methods:**

Four focus groups were conducted with community members (n= 73) in Norristown, PA and data were collected from Fall 2022 to Spring 2023. Open coding was used to conduct a thematic analysis of the transcript data.

**Results:**

Four themes were identified: 1) sources of concern, 2) lack of information surrounding lead, 3) systemic neglect, and 4) financial implications. Future research priorities include identifying contamination sources through testing of the household water supply, household paint, and soil as well as personal blood lead levels.

**Conclusions & Implications:**

There was broad community support for a lead exposure risk assessment that investigated soil, tap water, and paint sources of lead, as well as the prevalence of elevated blood lead levels in all generations. The study also highlighted the lack of information about lead exposure in non-English speaking communities, the need for providing language-appropriate information, and the necessity for multiple lead exposure assessment methods.

## Introduction

Lead is a toxic heavy metal and an environmental contaminant that disproportionately impacts communities of color across the United States [1], including members of racial-ethnic minority groups [2], economically and socially marginalized groups [3, 4], immigrants [4], and refugees [4]. According to Muller [1], these individuals are typically more likely to live in areas with aging infrastructure or homes built before 1978 with deteriorating lead-based paint and outdated plumbing, as well as in regions where past land use patterns increase exposure to contaminated soil. In 2014, increasing national attention was given to lead contamination due to the water crisis in Flint, Michigan, where the highest concentrations of lead were found in low-income communities [1, 5]. Children are at an increased risk of lead exposure due to their relatively rapid development, higher absorption rates, and more frequent hand-to-mouth activity [3, 6]. No blood lead level (BLL) is safe for children, as even low-level exposure causes harmful effects [1, 7]. Lead crosses the placental barrier, exposing fetuses in utero (LeBrón et al., 2019), and additional exposure can occur during infancy through breastfeeding or formula feeding through contaminated water [4, 8]. Once in the bloodstream, lead crosses the blood-brain barrier, substituting calcium ions [1, 9] and disrupting biological processes. It affects nearly every system of the body, especially the central nervous system, with irreversible effects [9]. Despite often being asymptomatic, even at high levels, lead poisoning can cause lasting damage [10], including cognitive impairments from prefrontal cerebral cortex, hippocampus, and cerebellum damage [9], and behavioral and intellectual deficits such as lower IQ [3, 8, 11], attention deficit and hyperactivity disorder [12], aggression [13], and underperformance in school [14]. In adults, lead exposure is linked to infertility [7], depression [15], eye disease [16], hypertension [17], and kidney dysfunction [17].

Recently, community based participatory research (CBPR) programs have been used to empower disenfranchised communities, including those which experience disproportionate exposure to environmental contamination [18, 19]. This approach includes researchers and community stakeholders as equal partners in all steps of the research process with the goals of educating, improving practice, and bringing about social change [20, 21]. While CBPR programs have been successful in engaging community stakeholders [22] and reducing lead exposure [23, 24], few to no studies have evaluated its effectiveness for reducing lead exposure in Latino communities. This is especially troublesome as Latino communities often face language barriers when communicating about threats to their health and wellbeing [25].

### • Local Context

Norristown, a municipality in Pennsylvania, located 15 miles northwest of Philadelphia, has a history of industrial activity, including factories, lumber yards, sawmills, and textile mills [26, 27], which contributed to environmental lead contamination [26, 27] 28]. Over time, waves of Latino immigrants arrived in Norristown, with a significant influx of Mexican immigrants occurring in the 21st century. The Latino population in Norristown grew from 828 individuals (2.7% of the population) in 1990, to 3,282 (10.5%) in 2000, and then to 9,700 (28.3%) in 2010 [27]. As of July 2023, Hispanics or Latinos make up 27.4% of Norristown’s population [29].

Norristown, PA has amongst the highest average child blood lead level in the State of Pennsylvania [30]. In Montgomery County, where Norristown is located, elevated BLL were more prevalent among Hispanic children (2.48% of Hispanic identifying children tested, no total number reported) compared to non-Hispanic White (0.41%) and non-Hispanic Black (1.04%) [30]. The risk of lead exposure is seen in other areas of the state, such as in Philadelphia after Hurricane Ida in 2021, where flooding introduced bio-accessible lead into homes [1]. These factors highlight the need to identify research priorities for a community driven lead exposure risk assessment in communities. Therefore, the purpose of this research was to establish research priorities for a community driven lead exposure assessment.

## Methods

The investigative team for this study consists of both academic and community-based investigators, collectively referred to as “the investigators.” The investigators all had connections with the Centro de Cultura, Arte, Trabajo, y Educación (CCATE), a non-profit organization in Norristown, PA that works to empower the Latino community through culture, art, career development, health programming, education and environmental stewardship. A shared goal of the investigators was to develop a research project addressing lead exposure within the Latino community. To determine the context for this study, the investigators held multiple working meetings to explore potential research pathways. They utilized a community-based participatory research (CBPR) framework, focusing on the domains of context and group dynamics [31]. Context refers to the social, historical, and structural factors influencing the overall CBPR process, while group dynamics encompass three sub-dimensions—individual, structural, and relational dynamics—that shape CBPR partnerships and contribute to diverse outcomes [31]. Although the domains of intervention and research and outcomes are outside the scope of this manuscript, the research priorities presented here align with the CBPR process as part of the larger, ongoing parent study, which is in the longitudinal data analysis and collection phase. This qualitative descriptive study relied on focus group interviews to collect data to answer the research question: What does the Norristown, PA Latino community know about lead risk and exposure?

### Focus Groups

Four focus groups were conducted in Fall 2022 and Spring 2023, each lasting 90 minutes. Participants were recruited via snowball sampling through community organizations. One recording was corrupted, leaving transcripts for three sessions. Three groups were in Spanish, and one was bilingual, reflecting participants’ cultural dynamics. The sessions were facilitated by bilingual author XX, with support from bilingual author YY. Reflexive praxis was employed throughout the process to ensure self-awareness and continuous learning. Transcripts were translated into English or Spanish as needed, ensuring accessibility for all investigators, as not all team members were bilingual.

## Thematic Analysis

The transcripts were then open coded by authors ZZ and YY, enhancing analytical rigor by incorporating diverse perspectives into the data analysis [32]. Each coder independently read the transcripts repeatedly, and assigned codes based on emerging themes. The coding team held weekly meetings to discuss and reconcile any differences in coding, ensuring consensus and enhancing the validity of the thematic analysis. All coding decisions were documented, and a codebook was developed and refined throughout the process. Final themes were derived through a collaborative analysis, and discrepancies were resolved through discussion between the coders.

## Results

Our analyses of the transcripts revealed four key themes: 1) sources of concern, 2) lack of information surrounding lead, 3) systemic neglect, and 4) financial implications. These four key themes, further divided into subthemes highlighting the Latino community’s research priorities. Quotes representative of each theme, in both English and Spanish, can be found in the supplemental material of this article.

## Theme 1: Sources of Concern

Various contamination sources were identified, both inside and outside of participants’ households. Potential sources of concern identified were tap water, soil in yards/gardens, and household paint. Participants expressed worry that they drank contaminated tap water which came in contaminated or became contaminated from old, corroded pipes, as well as floods which expose community members to lead-contaminated water and soil from the streets that enters their homes. A participant shared that Latino culture values homegrown food and herbs, but participants are unaware of their consumption of potentially contaminated food due to a lack of information in the community. Active dducation efforts by CCATE have been successful in teaching about lead contamination in soil. Through these educational sessions, community members learned the importance of adding organic materials, such as fertilizers and compost, to their soil. It was mentioned that many homes in Norristown built before 1978 contain lead-based paint, and participants spoke of a child’s curiosity which leads them to consume paint from picking and creating holes in walls that reach the layer despite overlying coats of paint.

## Theme 2: Lack of information surrounding lead

Participants identified that, as a community, they are at a disadvantaged because of the lack of information available to them in Spanish, especially in healthcare settings, even though they live in an area with a large Latino population. They also expressed a lack of information in various areas of knowledge, wanting to know more than the basics of what lead exposure is. Additionally, participants wanted to know of locations they can seek medical care without health insurance, including clinics. For example, a participant mentioned that as a community, many people do not go to a primary care provider because they do not have the resources, or they are fearful, highlighting the medical distrust many experience. Participants were also unaware if recreational spaces, such as parks and playgrounds were contaminated or historically contaminated, potentially putting children who play in these spaces at risk of touching contaminated surfaces. Community members wonder when they will have access to information regarding contaminated sites, and if it will be provided in Spanish.

## Theme 3: Systemic Neglect

The health and safety of the community was identified as a priority due to the negligence by major stakeholders, including workplaces, health care systems, and landlords. Workplaces failed to protect their employers by only distributing masks and goggles during inspections, especially in occupations such as remodeling and construction where they may be in contact with contaminated surfaces. Health care systems failed to provide lead exposure reduction information or materials to the family of children who were tested for lead exposure and identified as having a BLL slightly below the action level. Participants reported that in their community, blood lead testing was not universally conducted by pediatricians and health centers. Instead, they were completed at the preference of the physician providing care for their children. Thus, there is a discrepancy between providers screening for lead risk and the state required BLL testing for Children’s Health Insurance Program and Medicaid recipients and what is reported by families. Furthermore, landlords failed to show up when requested by the tenant and exhibit poor maintenance of buildings that go beyond surface level fixes; thus highlighting the poor power dynamic that may lead tenants to not express their concerns of lead as well as the aging infrastructure of Norristown that perpetuates lead exposure.

## Theme 4: Financial Implications

The financial implications of lead exposure were the community’s final priority. They feared losing their homes and subsequently finding replacement housing due to scarcity and expense. They feared being perceived as complainers when speaking out about their unsafe conditions because they could potentially lose their jobs and homes, without time to relocate or plan. A participant shared a story of an accident when a young man cut his hands while working because he was not given gloves, but he never said anything because he feared speaking out. Finally, the accumulation of costly expenses from water filtration systems were further mentioned as a barrier to reducing exposure risk. These costs include purchasing, installing, cleaning, and the replacing water filter systems.

## Discussion

The personal experiences and information shared by the Latino community in Norristown, PA highlight the continued issues surrounding increased lead exposure risk, leading to lead contamination and adverse health effects in racial and ethnic minority groups [1]. Participants revealed lead exposure pathways within their community and the lack of resources and information, especially in Spanish, to help them understand lead remediation and reduce future lead exposure.

### Sources of lead exposure in Norristown, PA

Sources of lead contamination identified were drinking water, soil, household paint, toys, and pottery. Regarding drinking water, the potential for corroded pipes, similar to those in Flint, Michigan, may pose a risk in Norristown as one participant noted pipe corrosion in their home [5]. In this study, Manayunk, an urban area with a large industrial past like Norristown only about 13 miles away, was detected to have greater concentrations of metals compared to East Falls, an urban area with less historical land use contributing the lead contamination [33]. With an increasing potential of flooding due to climate change [34], policy change is needed to eliminate the disproportionate presence of lead in low-income immigrant communities.

Culturally and linguistically congruent education sessions are crucial for increasing knowledge, mitigating lead exposure risk, and preventing misinformation. One participant’s belief that lead must be touched to be absorbed highlighted the need to clarify that lead can also be ingested and inhaled. Similar efforts have proven effective, such as educational programs for construction workers and families, which used plain language, visuals, interactive activities, and action plans to improve knowledge [35]. Community-focused education may be especially impactful for Latino communities, where multi-family and multi-generational households facilitate interpersonal learning [36]. CCATE, a trusted community hub, has fostered such learning since 2018 through participatory action research, actively disseminating research findings and fostering action through community organizing, conference presentations, and consultations with local representatives and institutions. Community-engaged activities should continue to be implemented, such as community gardens, as they improve the environment, community, and individual well-being by providing a space to garden, access to a green space, and a place for social interaction [37]. However, it is worth noting that community garden soil in Norristown, PA contained higher lead levels than surrounding locations [38]. Consequently, utilizing citizen science to conduct broader lead testing of the soil in the region may be beneficial.

Paint was the third contamination source identified as lead-based paint can peel, chip, or turn into dust, which children can then ingest or inhale (Muller et al., 2018). Participants spoke of the curiosity children have which puts them at an increased risk for lead contamination because that curiosity leads them to create holes in walls that reach the contaminated layer. Participants highlighted the value and widespread use of traditional Mexican kitchenware such as pots, pans, plates, and artifacts in many of their homes for drinking, eating, and decoration. Yet, these items can be contaminated with lead through the varnish base and paint used on the clay [39]. According to the Food and Drug Administration, lead could be found in the glazes or decorations and can leach into food or drink inside of the pottery if it is not manufactured properly by not being fired at a certain temperature and for a certain amount of time [40].

All three contamination sources (water, soil, and paint) are potential exposure pathways for the entire community of Norristown, affecting children and adults. To mitigate and prevent future lead exposure risk, household water, soil, and paint testing as well as personal blood lead testing are needed to identify the presence of lead and implement interventions to reduce the negative consequences associated with lead poisoning for people of all ages.

### Lack of information in Spanish and surrounding lead

Access to information in one’s own language is critical to understanding the information being presented, yet various participants spoke of the lack of lead exposure risk information available to them in Spanish, which for many is their first language, or the only language which they speak. In health care settings where participants would go to be tested for lead, they know they are not going to be heard, understood, and communicated to properly, potentially leaving more confused than when they entered. Participants may have experienced this lack of information because of the language barriers themselves, or they have heard stories from other members of their community. A previous study [41] highlighting the frustrating pediatric health care experiences of limited English proficiency (LEP) Latina mothers found six themes among the analysis of qualitative interview studies: 1) the “battle” of managing language barriers, 2) preference for bilingual providers, 3) negative bias toward interpreted encounters, 4) “getting by” with limited language skills, 5) fear of being a burden, and 6) stigma and discrimination [41]. LEP Latina mothers preferred attending clinics with bilingual providers even if it was less convenient because it improved understanding and removed the fear of being a burden and misinterpreted. Clinics were also preferred over doctors for their health care needs by participants of our study who lacked health insurance. Even with clinics being preferred, not all participants knew which clinics were available to them. Useful information that can be given to the community regarding clinics include those within close proximity, what services they provide, if they have Spanish speakers that can talk to the Latino population, and feedback from community members that have attended these locations. Culturally competent care is vital to reduce patients’ fear and discomfort associated with healthcare services, as well as access to language-appropriate information and resources [36].

The community’s drive to learn more and have access to lead exposure information is clear. They want to not just know what lead is but also how they can identify lead poisoning, learn about sources of lead exposure, and get education and resources to reduce their exposure. They want their questions answered so they can then share it with their families and their community, seen when a participant stated, “We are going to learn [the information about lead] together and that is beautiful we are going to learn it together and we are going to give solutions, solutions together.” Outside of the empowerment circle which CCATE provides, many Latino community members do not hear about certain topics or speak about them. It is through shared moments that community members are empowered to advocate for themselves and for others, such as actively asking their health care provider for a lead test. For a particular participant, the concept of requesting a lead test never occurred to her, but it was in this communal space where community members felt comfortable, asked each other questions, shared stories, and bounced ideas off of each other that they made aware and felt empowered to advocate for herself.

Identifying locations that have been previously contaminated was another important topic for the community because they feared not knowing which recreation spaces were unsafe for their children, and though they were aware there was a list from the Environmental Protection Agency, they did not know how to locate it and if it would be in Spanish. The lack of warnings and signs in recreation spaces added a level of uncertainty for parents because they were unaware if these spaces were contaminated. Participants also lacked information on active prevention and intervention strategies that went beyond knowledge on lead.

### Systemic Neglect of the Latino community’s health and safety

The health and safety of the Latino community was neglected by major stakeholders including workplaces, health care systems, and landlords. It was noted that companies only distribute personal protective equipment (PPE), including helmets, gloves, and masks, to employees during inspections. Additionally, workers are only provided helmets, which does not protect them from occupational lead exposure. Workplaces only take on the expenses when they know they are at risk of being seen as having unsafe practices during inspections. According to a study by Arcury et al. [42], which analyzed baseline interview information and daily logs of 89 North Carolinaresidential roofers that were all male, mostly Mexican, and spoke Spanish as their first language, 30–40% of participants never received safety training [42]. Also, only 65.9% of participants of the study reported being provided eye protection, 40.2% were provided hearing protection, 59.3% were provided cut-resistant gloves, 54.7% provided abrasion-resistant gloves, 81.4% provided hard hats, and 93.0% provided harnesses [42]. Employers are thus failing to provide PPE to their employees and provide safety training to prevent accidents. Occupations, such as working in the construction industry, were brought up by the participants as being of concern for occupational lead exposure, an inherent risk of the construction industry [43] that is only multiplied by the lack of proper PPE [44]. Participants also did not always feel comfortable speaking up about their unsafe conditions because of their language barrier and fear of losing their jobs. This finding resembles other asymmetrical power dynamics that have also been expressed by other immigrant workers [45–47].

According to participants, healthcare systems also neglected the health and safety of community members through the lack of distribution of resources, including information and lead tests. A mother shared how no resources or information were given to her, or recommendations for future blood lead tests. Healthcare practitioners should be aware of lead remediation resources and provide them to persons found to have elevated BLLs. These include prevention measures, such as encapsulation of lead paint in the home [48]; connection with community resources, such as the region specific pediatric environmental health specialty unit [49]; and prompt referral for retesting and chelation therapy if warranted [50]. Participants also spoke of lead BLL tests not being conducted in all pediatric centers or clinics. This finding is contradictory to the literature that Hispanic individuals are more likely to receive blood lead level testing than other demographic groups [51, 52]. There were known decreases in the rates of BLL testing during the pandemic [53], and it is possible that this effect was being felt by community members. Further work is therefore needed to understand the local context of blood lead level testing for Latinos living in the Norristown region.

Many people rent in Norristown, and it was noted that landlords do not care enough to maintain their properties surface level fixes or help the people living in them. A participant stated, “We have heard stories of people who call their landlords, but they do not show up,” which can discourage tenants from contacting their landlords if they have any problems concerning lead exposure. Similarly, low-income renters of color in Albuquerque, NM, a predominantly American Indian/Alaskan Native, Latino, and White area, expressed the non-responsive management of landlords, housing quality issues, and racism associated with housing [54].

### Financial burden associated with lead contamination

The financial implications of lead exposure were the community’s final priority. Housing insecurity or concerns about housing instability led community members to be fearful of losing their homes, and subsequent replacement housing due to scarcity and expense. These finiacial barriers could lead to them not moving out of potentially unsafe homes with poor maintenance by landlords [36]. Schmidt has coined this phenomenon as “negotiating neglect” in which low-income renters who are unable/unwilling to move endure the consequences of housing in disrepair including decision making around requesting repairs from a landlord, investing of personal finances into a rented dwelling, and the impacts of such chronic stress on their health and wellbeing [55]. It is worth noting that in relation to lead remediation, for every $1.00 invested on lead remediation there is a $2.60 return on investment [56].

Not only did participants discuss their environmental exposures at home, but they were also concerned about potential lead exposure at work. While we did not specifically ask about participant occupations, they spoke of their concerns during the focus groups. Occupations at increased risk for lead exposure include construction workers and metal foundry workers [43, 57]. One participant disclosed of an incident at his workplace in which the company was not providing appropriate PPE and there was fear of speaking up due to worry about loss of job and subsequent income. It is well established that proper PPE reduces many occupational exposures, including to heavy metals, such as lead [58, 59]. This indicates that if workers are not provided masks or gloves when working with lead contaminated surfaces, they may fear speaking up as well. Latino communities also face barriers including financial instability [36], high rates of uninsurance [36, 60], lack of PPE [36, 61], and confusion regarding their employee protections [36] leaving them vulnerable to losing their homes and job. Participants also mentioned the burden of costly interventions to reduce lead exposure. Despite their desire to reduce exposure, financial barriers may prevent action. It is thus important to provide community members with low-cost, accessible interventions to reduce the burden of lead mitigation.

### Limitations

Study limitations include losing one of the recordings from a focus group due to technology issues. We were only able to transcribe and analyze three of the four focus groups which were conducted, meaning that about 1 hour of discussion by the community was lost. Additionally, while we attempted to invite a diverse sample of Norristown residents to our study, these research priorities were largely identified by the Spanish-speaking Latino community. Insights from other marginalized groups could potentially increase the robustness of this work. Further, qualitative findings are not generalizable and there is a risk of focus group participants providing socially desirable answers.

## Conclusions and Final Recommendations to Eliminate Health Inequities

The study findings provide insight to the needs of the community related to lead exposure, risk and screening. Participants’ narratives reveal inequities in housing, employment, healthcare, and environmental safety, compounded by language barriers and lack of culturally tailored resources. This underscores the necessity of addressing lead exposure as a multidimensional public health challenge requiring collaboration across individual, community, and policy levels. We recommend that the following occur to help eliminate health inequities from environmental exposures:

- Partner with trusted community organizations to provide culturally and linguistically appropriate education and resources in Spanish. This should include practical strategies for reducing lead exposure (e.g., soil amendments for gardening) and fostering communal learning spaces to empower residents.
- Advocate for stronger enforcement of housing and workplace safety regulations, provide resources for landlords to maintain lead-free properties, and ensure healthcare providers comply with laws requiring pediatric blood lead testing.
- Develop accessible, multilingual resources to educate the community about lead exposure risks, prevention, and available healthcare options. Include actionable guidance on navigating systems like clinics, lead testing facilities, and government resources.

## Data Availability

Transcript data are available upon reasonable request, but have not been made openly accessible due to privacy concerns.
